# Exploring Key Biomarkers and Common Pathogenesis of Seven Digestive System Cancers and Their Correlation with COVID-19

**DOI:** 10.3390/cimb45070349

**Published:** 2023-06-30

**Authors:** Zuming Xiong, Yongjun Yang, Wenxin Li, Yirong Lin, Wei Huang, Sen Zhang

**Affiliations:** Department of Colorectal and Anal Surgery, The First Affiliated Hospital of Guangxi Medical University, Nanning 530021, China

**Keywords:** digestive system cancers, COVID-19, common biomarkers, pathogenesis, diagnosis, prognosis

## Abstract

Digestive system cancer and COVID-19 significantly affect the digestive system, but the mechanism of interaction between COVID-19 and the digestive system cancers has not been fully elucidated. We downloaded the gene expression of COVID-19 and seven digestive system cancers (oral, esophageal, gastric, colorectal, hepatocellular, bile duct, pancreatic) from GEO and identified hub differentially expressed genes. Multiple verifications, diagnostic efficacy, prognostic analysis, functional enrichment and related transcription factors of hub genes were explored. We identified 23 common DEGs for subsequent analysis. CytoHubba identified nine hub genes (*CCNA2*, *CCNB1*, *CDKN3*, *ECT2*, *KIF14*, *KIF20A*, *KIF4A*, *NEK2*, *TTK*). TCGA and GEO data validated the expression and excellent diagnostic and prognostic ability of hub genes. Functional analysis revealed that the processes of cell division and the cell cycle were essential in COVID-19 and digestive system cancers. Furthermore, six related transcription factors (E2F1, E2F3, E2F4, MYC, TP53, YBX1) were involved in hub gene regulation. Via in vitro experiments, *CCNA2*, *CCNB1*, and *MYC* expression was verified in 25 colorectal cancer tissue pairs. Our study revealed the key biomarks and common pathogenesis of digestive system cancers and COVID-19. These may provide new ideas for further mechanistic research.

## 1. Introduction

With lifestyle changes, digestive system cancer incidence and mortality are increasing gradually worldwide, where digestive system cancers significantly harm human health [1]. Statistically, digestive system cancer incidence comprises ~30% of cancer cases worldwide and ~40% of deaths related to cancer worldwide [2]. The most recent US data stated that approximately 348,840 new digestive system cancer cases and 172,010 new digestive system cancer deaths may occur in the US in 2023 [3].

While different digestive system cancers demonstrate apparent heterogeneity, their occurrence and development are closely related to the human immune system, digestive tract microorganisms, and dietary habits [4,5]. These findings suggested that there may be common developmental mechanisms and biological links among digestive system cancers. Research statistics [6,7] determined that the common digestive system cancers are oral cancer (OC) [8], esophageal cancer (ESC) [9], gastric cancer (GC) [10], colorectal cancer (CRC) [11], hepatocellular cancer (HCC) [12], bile duct carcinoma (BDC) [13], and pancreatic cancer (PC) [14]. The underlying molecular regulatory mechanisms shared by these seven digestive system cancers must be explored further.

Since the first case of pneumonia of unknown cause was reported in December 2019, severe acute respiratory syndrome coronavirus 2 (SARS-CoV-2) has spread rapidly worldwide, posing a significant threat to human health. Severe COVID-19 might also be accompanied by various complications, such as pneumonia, acute respiratory distress syndrome, and sepsis [15], which might be related to the immune dysfunction and cytokine storm caused by COVID-19 [16,17]. Simultaneously, many studies reported that many patients infected with the SARS-CoV-2 also have gastrointestinal symptoms, which include anorexia, nausea, vomiting, diarrhea, and abdominal pain. These patients face a greater chance of developing severe disease, and they might face a worse outcome and higher risk of death [18]. Further investigations determined that the novel coronavirus mainly acts on ACE2 receptor-positive cells, where ACE2 receptor expression is highest in intestinal cells, including the small intestine, colon, and duodenum. Compared with the lung, the digestive organs have higher ACE2 expression levels [19]. These findings indicated that in addition to intestinal inflammation caused by the systemic inflammatory response due to SARS-CoV-2, the virus may also directly affect the digestive system by attacking intestinal cells [20].

A 2023 study noted that COVID-19 infection can also lead to long-term coronavirus sequelae. This debilitating post-infection multisystem disease impairs the ability to perform daily activities [21]. The gastrointestinal tract is also one of the main sites affected by the long-term sequelae of COVID-19, and the acute-phase immune system disorder will persist in the sequelae phase [22,23]. At the same time, COVID-19 sequelae can affect the survival of patients with cancer and cancer treatment continuity [24,25].

The above evidence indicated that the digestive system is one of the main sites of the novel coronavirus infection. Considering the long-term immune system effects of the novel coronavirus, digestive diseases, specifically digestive system cancers, might be closely related to the novel coronavirus [26]. Therefore, their potential common molecular action mechanism warrants further discussion.

Therefore, the study objective was to ascertain common hub genes between seven digestive system cancers and SARS-CoV-2 infection through multi-dimensional analysis and explore their molecular action mechanisms. We also analyzed the diagnostic, prognostic, transcription factors, and co-expressed genes of hub genes. Then, the expression trend of these hub genes was verified in several gene data sets. The common hub genes of the seven digestive system cancers and COVID-19 may provide new insights into the biological mechanisms of these eight diseases.

## 2. Materials and Methods

### 2.1. Data Source

GSE30784 (OC), GSE44021 (ESC), GSE29272 (GC), GSE106582 (CRC), GSE102079 (HCC), GSE26566 (BDC), and GSE62165 (PC) were used as the training sets. We downloaded these datasets from the database of Gene Expression Omnibus (GEO) [27] (http://www.ncbi.nlm.nih.gov/geo, accessed on 1 December 2022).

The first batch of test sets came from TCGA TPM (transcripts per million) and was analyzed with Xiantao online tools (https://www.xiantaozi.com/, accessed on 1 December 2022). As very little normal tissue matches pancreatic cancer, TCGA+GTEX was used instead. The Cancer Genome Atlas (TCGA) data of the Xiantao online tools were updated in August 2022.

GSE37991 (OC), GSE96668 (ESC+GC), GSE66229 (GC), GSE44076 (CRC), GSE112790 (HCC), GSE60979 (BDC+PC), and GSE15471 (PC) were used as the second batch of test sets. The GEO datasets were converted and annotated by Perl or R software.

### 2.2. Identification of Differentially Expressed Genes (DEGs)

We obtained the DEGs in the datasets of GEO by the R packages limma or DESeq2. Only the genes with adjusted *p* < 0.05 and |logFC| ≥ 0.585 were identified as DEGs. The R packages VennDiagram and plotrix were used to obtain the intersecting upregulated or downregulated DEGs of the eight diseases, which were identified as common DEGs.

### 2.3. Gene Ontology (GO), Kyoto Encyclopedia of Genes and Genomes (KEGG), and Protein–Protein Interaction (PPI) Network Construction of Common DEGs

The enrichment of the common DEGs were analyzed with KEGG and GO using the R package clusterProfiler to identify the functions, biological pathways, and cell location [28,29,30]. *p* < 0.05 was considered a significant result. The enrichment analysis results were visualized with the R packages enrichplot and ggplot2.

STRING is a Global Biodata Coalition and ELIXIR Core Data Resource. It is utilized to determine the relationship between genes or proteins of interest [31] (STRING: https://cn.string-db.org,version 11.5, accessed on 13 March 2023). Interactions with a combined score >0.4 were considered statistically significant. The PPI network was visualized with Cytoscape 3.9.1.

### 2.4. Selection and Analysis of Hub Genes

The hub genes were identified using the Cytoscape cytoHubba plug-in. Here, we used common algorithms of degree to evaluate and select nine hub genes. Subsequently, we constructed a network of the co-expression of these nine hub genes using GeneMANIA (http://www.genemania.org/, accessed on 21 February 2023). GeneMANIA finds other genes that are related to a set of input genes. These genes have association data, including protein and genetic interactions, pathways, co-expression, co-localization, and protein domain similarity. Finally, the KEGG and GO analyses of the hub genes were conducted.

### 2.5. Validation of Hub Genes in TCGA and GEO Datasets of Seven Cancers

The expression and diagnostic receiver operating characteristic (ROC) of nine hub genes were verified in TCGA datasets (first batch of test sets). Then, the area under the ROC curves (AUC) was calculated to represent the diagnostic value of the expression level. These analyses were conducted through the Xiantao online ROC curves. The expression of the nine hub genes was also verified in another seven GEO datasets (second batch of test sets). *p* < 0.05 was considered significant.

### 2.6. Hub Gene Blood Expression and Diagnostic Value in HCC and COVID-19

The expression levels of four GEO datasets were assessed through the R packages limma or DESeq2. The differential expression of these hub genes was examined between normal and HCC (GSE114564), hepatitis and HCC (GSE114564), and normal and COVID-19 in GSE171110 and GSE152418. The independent diagnostic efficiency of the nine hub genes was presented in the aforementioned datasets. The ROC diagnostic model efficiency of the hub genes in blood was also determined.

### 2.7. Hub Gene Prognostic Value in OC, HCC, and PC

To examine the correlation between the nine hub genes and prognosis, we used Kaplan–Meier to assess the overall survival (OS) between high- and low-expression groups in OC, PC, and HCC. The analyses were performed on Xiantao online tools (auto select best truncation value).

### 2.8. Transcription Factors Predicting Hub Genes and Verification in TCGA

Transcriptional Regulatory Relationships Unraveled by Sentence-based Text (TRRUST) mining (https://www.grnpedia.org/trrust/, accessed on 21 February 2023) is a database for predicting transcriptional regulatory networks containing the target genes corresponding to transcription factors and the inter-transcription factor regulatory relationships [32].

The transcription factors that regulate the hub genes were obtained through the TRRUST database, and an adjusted *p* < 0.05 was considered significant. Subsequently, we verified the expression levels of these transcription factors in TCGA datasets of the seven cancers on the Xiantao online tool.

### 2.9. qRT-PCR

CRC tissues and corresponding adjacent colorectal tissues were obtained from the First Affiliated Hospital of Guangxi Medical University between April 2022 and September 2022. A total of 25 pairs of tissues were analyzed. The patients from whom the tissues were obtained underwent surgery at our hospital. All patients provided their written informed consent before tissue collection. The study was approved by the First Affiliated Hospital of Guangxi Medical University ethics committee.

Inclusion criteria: 1. Age older than 18 years old; 2. Patients diagnosed with colorectal cancer for the first time and undergoing surgical resection; 3. The postoperative pathological specimen was diagnosed as colorectal adenocarcinoma by two pathologists. Exclusion criteria; 1. Patients who refused to participate in this study; 2. Patients with postoperative pathological diagnosis of non adenocarcinoma.

All samples were stored at −80 °C until use. Total RNA was extracted with TRIzol (No. B511321; Sangon Biotech, Shanghai, China). Complementary DNA (cDNA) was synthesized by a SweScript RT II First Strand cDNA Synthesis Kit (No. G3333, Servicebio, Wuhan, China). Gene expression was measured using 2× Universal Blue SYBR Green qPCR Master Mix (No. G3326, Servicebio). Relative gene expression was normalized through GAPDH and calculated by the comparative threshold cycle (2^−ΔΔCt^) method.

The following primer sequences were used: *GAPDH*, forward: GGAAGCTTGTCATCAATGGAAATC and reverse: TGATGACCCTTTTGGCTCCC; *CCNA2*, forward: AGTAAGACTGGCATCCAAGAAGT and reverse: GGTTTGCTCTCTGGTTTTACTCT. *CCNB1*, forward: AATAAGGCGAAGATCAACATGGC and reverse: TTTGTTACCAATGTCCCCAAGAG. *MYC*, forward: GTCAAGAGGCGAACACACAAC and reverse: TTGGACGGACAGGATGTATGC.

### 2.10. Statistical Analysis

The statistical analysis was performed using R 4.2.3. *p* < 0.05 was considered statistically significant.

## 3. Results

### 3.1. Identification of Common DEGs in Training Sets of Seven Cancers and COVID-19

**Figure 1:**
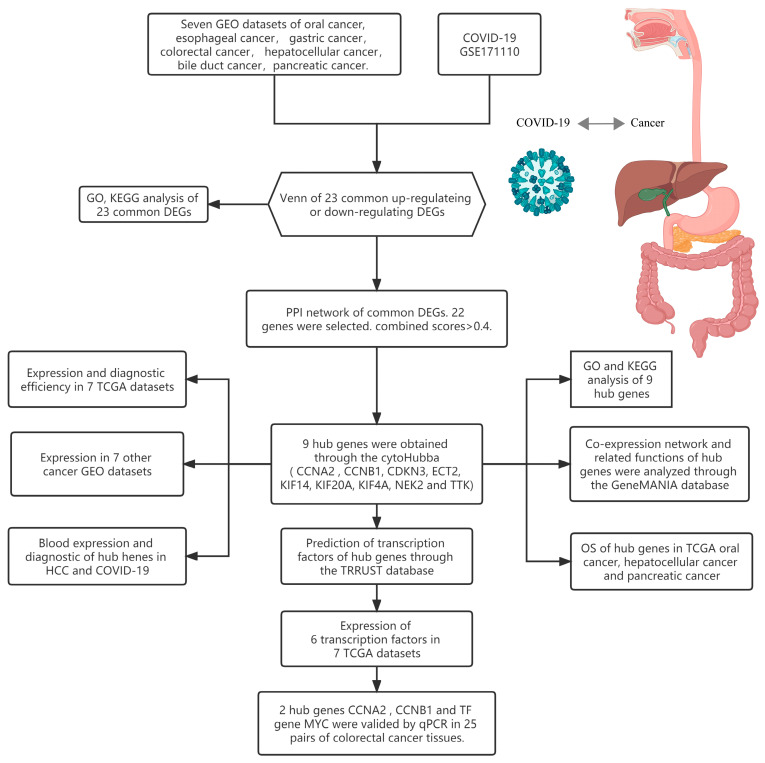
Research design flow chart.

**Figure 2:**
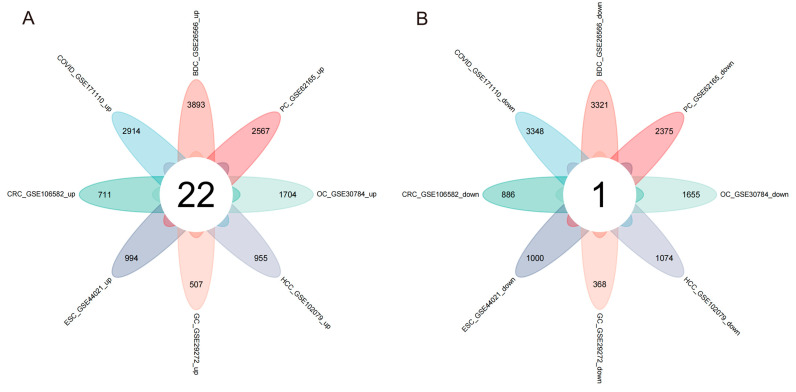
Venn diagram of upregulated and downregulated common DEGs in the eight diseases. (**A**) Twenty-two overlapping upregulated DEGs. (**B**) One overlapping downregulated DEG.

Figure 1 depicts the main steps of the study. Table 1 presents the basic information of the eight training datasets. After standardizing the GEO dataset gene expression, we identified the DEGs (3359 in GSE30784, 1994 in GSE44021, 875 in GSE29272, 1597 in GSE106582, 2029 in GSE102079, 7214 in GSE26566, 4942 in GSE62165, 6262 in GSE171110) and obtained 23 common DEGs (Figure 2A,B) (22 upregulated genes and 1 downregulated gene) between the seven digestive system cancers and COVID-19 (Table 2).

### 3.2. GO, KEGG, and PPI Network of Common DEGs

**Figure 3:**
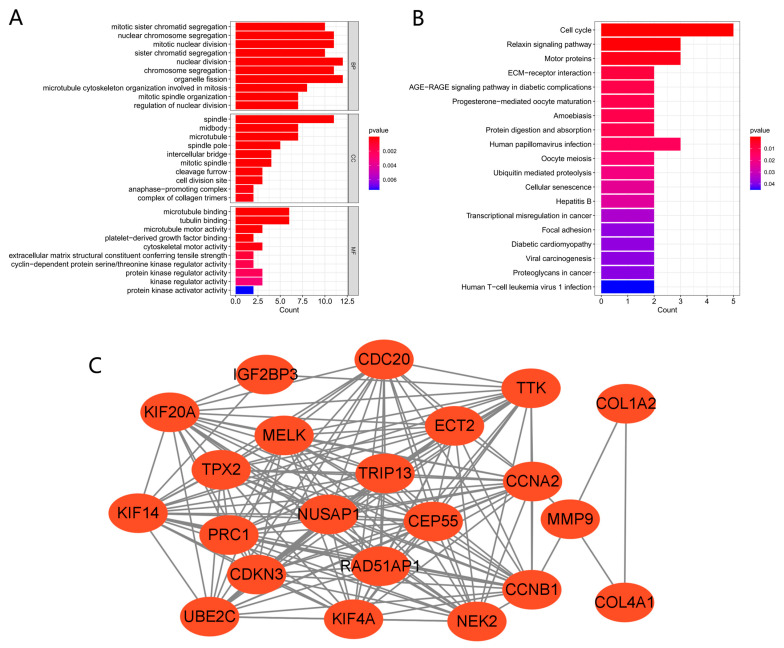
GO, KEGG, and PPI network of common DEGs. (**A**,**B**) KEGG and GO pathway enrichment analysis results. *p* < 0.05 was considered significant. (**C**) PPI network. Red indicates upregulated genes.

The biological functions and pathways among the 23 common DEGs were analyzed with the KEGG pathway and GO analyses (Figure 3A,B). GO analysis demonstrated that the genes were enriched in many biological processes (nuclear division, organelle fission, nuclear chromosome segregation) and molecular functions (microtubule binding, tubulin binding, microtubule motor activity). KEGG analysis demonstrated enrichment for cell cycle, Relaxin signaling pathway, human papillomavirus infection, hepatitis B, viral carcinogenesis, human T-cell leukemia virus 1 infection, and other pathways. These findings suggested that cell cycle, genetic material changes, and virus might be essential in the seven cancers and COVID-19.

A PPI network of the shared DEGs with combined scores >0.4 was constructed using Cytoscape, where the result contained 22 nodes and 161 interaction pairs (Figure 3C). These PPI network genes were all upregulated in the seven cancers and COVID-19.

### 3.3. Establishment, GeneMANIA, GO, and KEGG Analysis of Hub Genes

**Figure 4:**
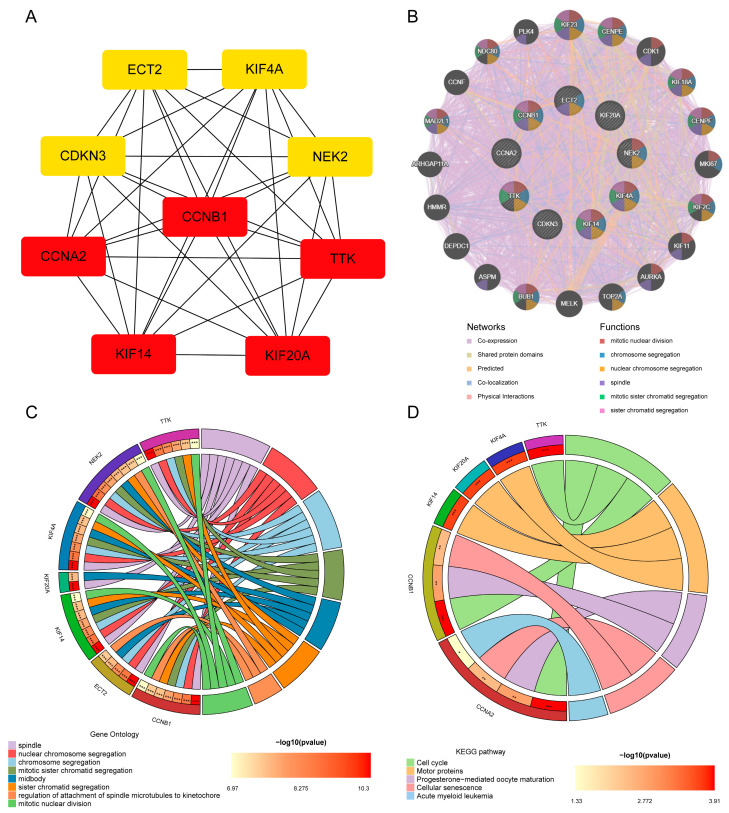
Hub gene analysis. (**A**) Network of PPI of the top nine hub genes from cytoHubba. (**B**) Hub genes and their co-expression genes were analyzed via GeneMANIA. (**C**,**D**) Analysis by GO and KEGG of hub gene enrichment. (* *p* < 0.05; ** *p* < 0.01; *** *p* < 0.001).

CytoHubba identified nine common hub genes (*CCNA2*, *CCNB1*, *CDKN3*, *ECT2*, *KIF14*, *KIF20A*, *KIF4A*, *NEK2*, *TTK*) (Figure 4A). These nine genes were all upregulated in the seven cancers and COVID-19. We analyzed the co-expression networks and related functions of these genes based on the GeneMANIA database. These genes yielded a complex PPI network with 83.18% co-expression, 7.48% shared protein domains, 4.71% predicted, 2.65% co-localization, and 1.99% physical interactions (Figure 4B).

GO examination demonstrated that these genes are primarily involved in the spindle, nuclear chromosome segregation, chromosome segregation, mitotic sister chromatid segregation, midbody, sister chromatid segregation, regulation of attachment of spindle microtubules to the kinetochore, and mitotic nuclear division (Figure 4C). Furthermore, KEGG pathway analysis demonstrated that they are mainly involved in the cell cycle, motor proteins, progesterone-mediated oocyte maturation, cellular senescence, and acute myeloid leukemia (Figure 4D). These results re-emphasize the importance of cell cycle and genetic material changes in the seven cancers and COVID-19.

### 3.4. Hub Gene Expression Validation and Diagnostic Efficiency in TCGA Datasets

**Figure 5:**
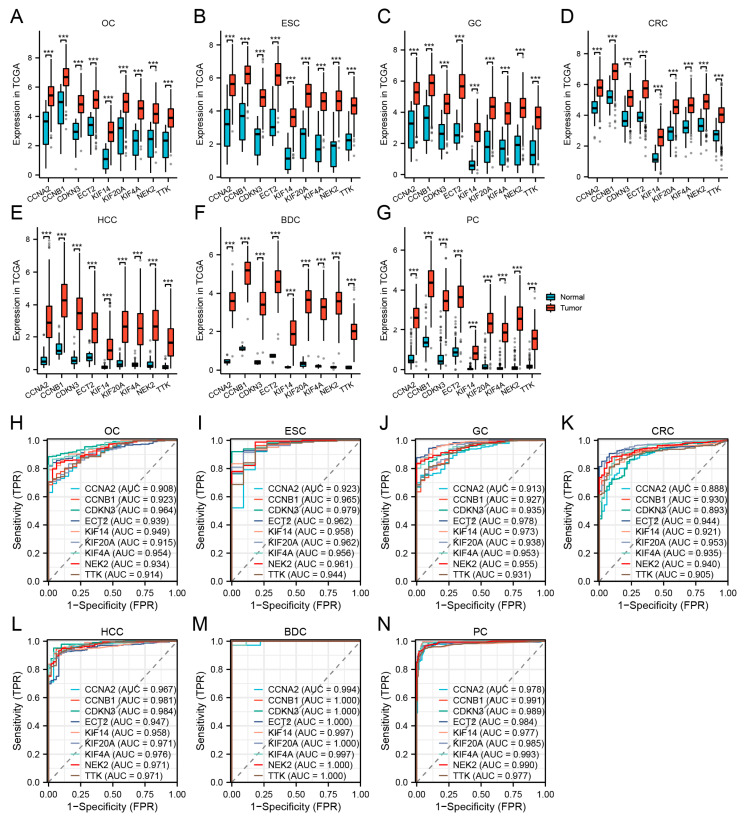
Validation of hub genes in TCGA. (**A**–**G**) Hub gene expression levels of OC (**A**), ESC (**B**), GC (**C**), CRC (**D**), HCC (**E**), BDC (**F**), and PC (**G**) in TCGA cohorts. (**H**–**N)** Diagnostic AUCs in seven TCGA cohorts. (*** *p* < 0.001).

To verify the reliability of the levels of expression of these hub genes, we examined their levels of expression in seven digestive system cancers from TCGA datasets. Table 3 depicts the basic data information of the seven TCGA datasets. In comparison with normal tissues, the hub genes were all significantly upregulated in cancer (Figure 5A–G). The AUC demonstrated that the nine hub genes had excellent diagnostic efficiency in the seven digestive system cancers (AUC: OC ≥ 0.914, ESC ≥ 0.923, GC ≥ 0.913, CRC ≥ 0.888, HCC ≥ 0.947, BDC ≥ 0.994, PC ≥ 0.984, Figure 5H–N).

### 3.5. Secondary Validation of Hub Gene Expression in Seven Cancer GEO Datasets

**Figure 6:**
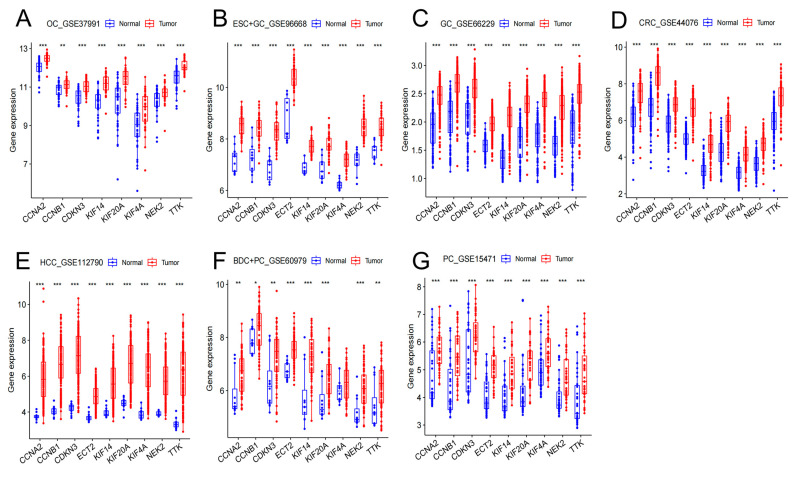
Hub gene expression levels in GSE37991 (**A**), GSE96668 (**B**), GSE66229 (**C**), GSE44076 (**D**), GSE112790 (**E**), GSE60979 (**F**), GSE15471 (**G**). (* *p* < 0.05; ** *p* < 0.01; *** *p* < 0.001).

The nine hub genes were validated again in GSE37991 (OC), GSE96668 (ESC+GC), GSE66229 (GC), GSE44076 (CRC), GSE112790 (HCC), GSE60979 (BDC+PC), and GSE15471 (PC). Table 4 lists the basic data information of the seven cancer test sets. Similarly, the high expression of hub genes was confirmed in the seven digestive system cancers (Figure 6A–G).

### 3.6. Blood Diagnostic Value of Hub Genes in HCC and COVID-19

**Figure 7:**
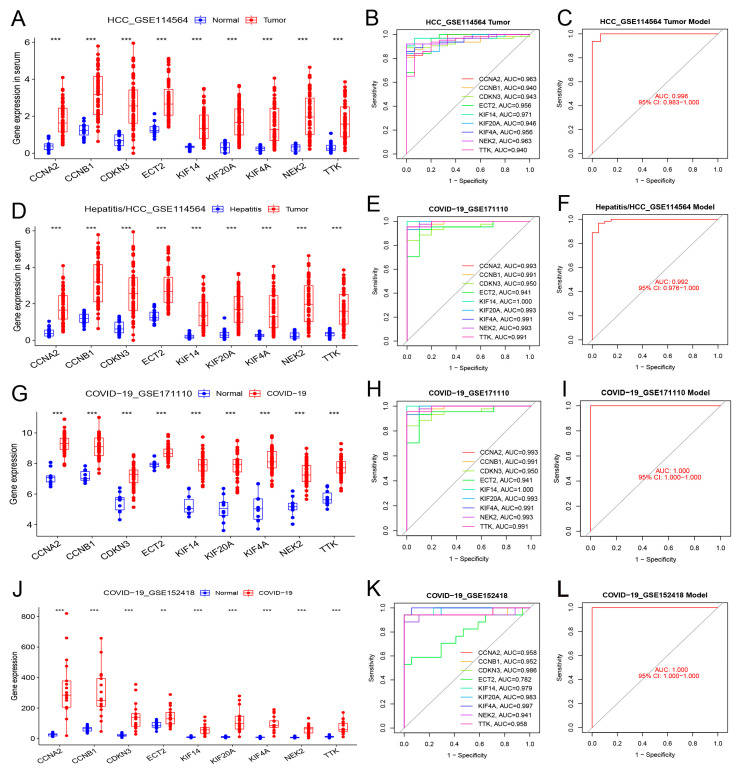
Differential expression, independent diagnostic efficiency, and receiver operating characteristic (ROC) diagnostic model efficiency of hub genes in the blood of patients with HCC and COVID-19. (**A**–**C**) Differential expression, diagnostic efficiency, and ROC diagnostic model between normal tissue and HCC (GSE114564). (**D**–**F**) Differential expression, diagnostic efficiency, and ROC diagnostic model between hepatitis and HCC (GSE114564). (**G**–**I**) Differential expression, diagnostic efficiency, and ROC diagnostic model between normal tissue and severe COVID-19 (GSE171110). (**J**–**L**) Differential expression, diagnostic efficiency, and ROC diagnostic model between normal tissue and COVID-19 (GSE152418). (** *p* < 0.01; *** *p* < 0.001).

Three datasets were used for verification to evaluate the value of the nine hub genes in blood. Table 5 presents the basic data of the blood datasets. The nine hub genes in the blood of patients with HCC exhibited the same upregulating trend as that of the samples between healthy people and those with hepatitis (Figure 7A,D). Blood testing was able to distinguish HCC from healthy people or those with hepatitis (AUC ≥ 0.940, Figure 7B,E).

In two COVID-19 datasets, the nine hub genes were significantly upregulated in COVID-19 patients (Figure 7G,J). These hub genes demonstrated excellent diagnostic value for severe COVID-19 (AUC ≥ 0.941, GSE171110, Figure 7H). The genes also demonstrated high diagnostic values for different severities of COVID-19 (AUC ≥ 0.782, GSE152418, Figure 7K). The model composed of these nine hub genes demonstrated excellent diagnostic value for HCC and COVID-19 (AUC ≥ 0.992; Figure 7C,F,I,L).

### 3.7. Hub Gene Prognostic Value in OC, HCC, and PC

**Figure 8:**
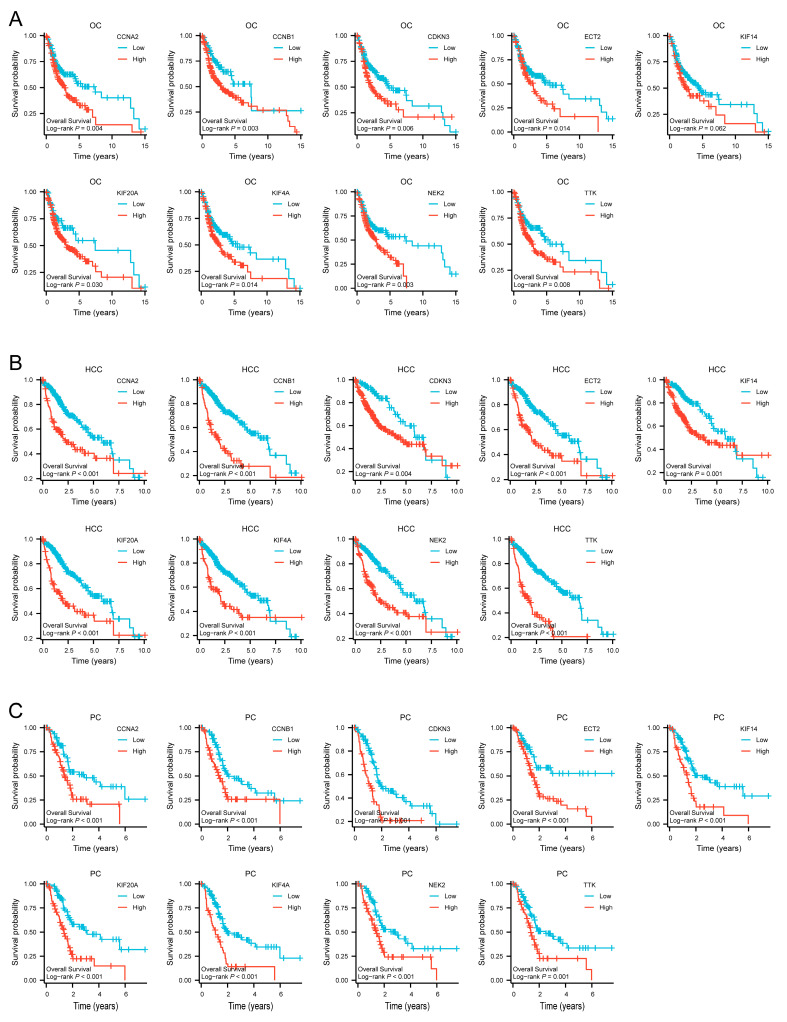
Survival curves of *CCNA2*, *CCNB1*, *CDKN3*, *ECT2*, *KIF14*, *KIF20A*, *KIF4A*, *NEK2* and *TTK* in TCGA cohorts. (**A**) Survival curves of hub genes in OC. (**B**) Survival curves of hub genes in HCC. (**C**) Survival curves of hub genes in PC.

The Kaplan–Meier plots of the nine hub genes demonstrated that higher expression levels indicated poorer OS in TCGA OC, HCC, and PC (Figure 8A–C). These results demonstrated that the hub genes *CCNA2*, *CCNB1*, *CDKN3*, *ECT2*, *KIF14*, *KIF20A*, *KIF4A*, *NEK2*, and *TTK* could predict OS in the OC, HCC, and PC cohorts. This finding further confirmed the value of the nine hub genes.

### 3.8. Prediction of Transcription Factors and Verification in TCGA Cohorts

**Figure 9:**
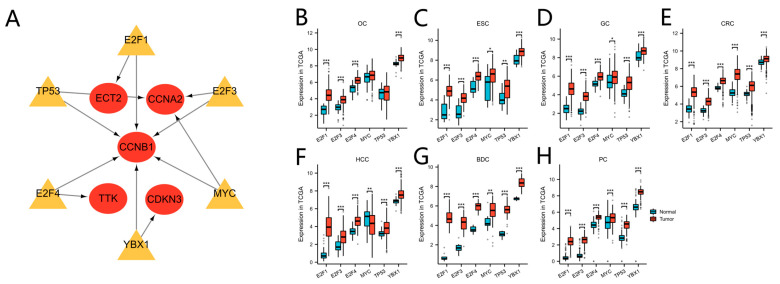
Transcription factor regulation of hub genes and transcription factor expression level in TCGA. (**A**) The regulatory network between the transcription factors and hub genes. Yellow indicates transcription factors, red indicates the hub genes. (**B**–**H**) The transcription factor expression levels between tumor and normal tissue in TCGA OC (**B**), ESC (**C**), GC (**D**), CRC (**E**), HCC (**F**), BDC (**G**), and PC (**H**) cohorts. (* *p* < 0.05; ** *p* < 0.01; *** *p* < 0.001).

Based on the TRRUST database, we determined that the transcription factors E2F1, E2F3, E2F4, MYC, TP53, and YBX1 might regulate the expression of the nine hub genes (Table 6 and Figure 9A). Verification revealed that the six transcription factors were almost upregulated in TCGA tumor samples of the seven cancers (Figure 9B,C). As the core regulated gene, *CCNB1* was regulated by the six transcription factors simultaneously.

### 3.9. qPCR Validation in Paired Colorectal Tissues

**Figure 10:**
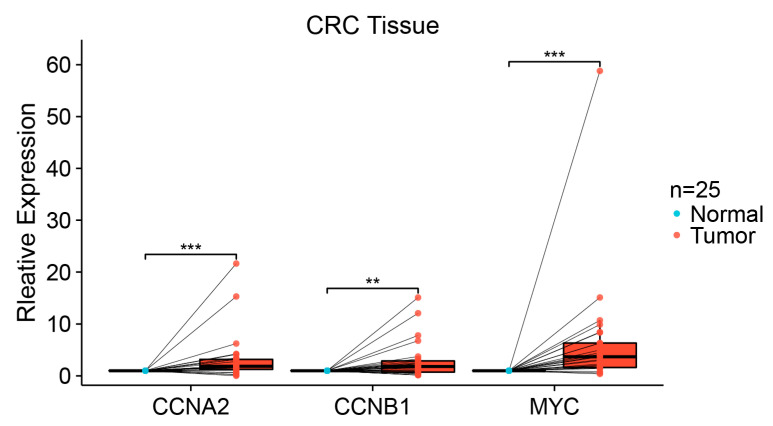
qPCR expression level of *CCNA2*, *CCNB1*, and *MYC* in 25 pairs of colorectal tissues. Each dot represents the expression of normal or CRC sample. (** *p* < 0.01; *** *p* < 0.001).

Two hub genes (*CCNA2*, *CCNB1*) and a transcription factor gene (*MYC*) were validated by qPCR in 25 pairs of CRC and adjacent normal colorectal tissues. The expression of the three genes was analyzed with the Wilcoxon signed rank test. The results demonstrated that *CCNA2*, *CCNB1*, and *MYC* expression was significantly elevated in CRC tissues compared to the paired normal tissues (*p* < 0.05) (Figure 10).

## 4. Discussion

As a whole continuous organ, the digestive system greatly influences the human body. Food interacts with the digestive organs as it moves from the mouth to the body. Various factors cause cancer, including diet, infection, immunity, environment, and genetic mutations. In this study, we explored the common hub genes of COVID-19 and seven digestive cancers and analyzed the gene function and interaction in depth. Our analysis results show that there may indeed be a potential common molecular mechanism between COVID-19 and digestive system cancer. Considering the affinity of COVID-19 to the digestive system, this indicates that the imbalance in gene expression caused by COVID-19 infection may have a certain impact on the incidence rate of digestive system cancer, which requires further research.

In this study, we identified 23 DEGs as co-regulatory genes of seven digestive cancers and COVID-19. Among these DEGs, nine genes (*CCNA2*, *CCNB1*, *CDKN3*, *ECT2*, *KIF14*, *KIF20A*, *KIF4A*, *NEK2*, *TTK*) were identified as hub genes. We determined that six transcription factors (E2F1, E2F3, E2F4, MYC, TP53, YBX1) participated in the transcriptional regulation of hub genes. The hub gene expression trend was highly consistent in the seven digestive system cancers and COVID-19 in the GEO and TCGA gene expression profiles, and there was high expression of most of the genes in the eight diseases. Due to the special reasons for bile duct and esophageal cancer surgery, it is difficult to obtain more normal samples. When there are fewer normal samples, this will have a certain impact on the accuracy of AUC. We included TCGA and multiple GEO for analysis, hoping to minimize the impact of errors as much as possible. Most of the diagnostic efficacies AUCs of the nine hub genes were >0.9, which was closely related to the OC, HCC, and PC prognoses. In HCC in particular, the hub genes exhibited excellent diagnostic value in the blood and tissues, and could also predict the OS of patients with HCC. In vitro, the *CCNA2*, *CCNB1*, and *MYC* expression trends were consistent with the bioinformatics analysis results, and the gene expression level differences were statistically significant.

Analyses of functional enrichment and pathways aid in an understanding of the essential hub gene biological processes and mechanisms in the human body. The analysis of KEGG pathway enrichment demonstrated that the hub genes were closely related to the cell cycle, motor proteins, progesterone-mediated oocyte maturation, cellular senescence, and acute myeloid leukemia. SARS-CoV-2 infection promoted the generation of multiple cytokines, the shutdown of mitotic kinases, and affected cell division and led to cell cycle arrest [33]. In addition, SARS-CoV-2 can induce cell senescence like other viruses and exacerbate the senescence-associated secretory phenotype (SASP), which is composed of pro-inflammatory factors, extracellular matrix degradation factors, complement activators, and pro-coagulation factors secreted by aging cells [34].

Analysis of the enrichment of GO demonstrated that hub the genes were mainly located in the intracellular chromatin and microtubules, participated in biological processes, e.g., chromosome separation and microtubule composition, and were also linked to the activity of intracellular microtubule movement, motion-related proteins, and protein kinase regulation. Microtubules are composed of tubulin and exist in the cytoplasm. They are closely related to cell movement, signal transduction, and cell division, and participate in the formation of spindles, grana, centrioles. Motor proteins, microtubules, and other tubulin and actin structures are vital to cancer cell proliferation and invasion [35]. Even et al. demonstrated that interfering with microtubule formation promoted CRC onset and CRC cell migration by disrupting apical cell polarity, epithelial formation, proliferation, apoptosis, mitotic spindle dynamics, and cell cycle progression [36]. Protein kinases are critical regulators of phosphorylation-based signal transduction and share structurally similar catalytic domains critical to protein binding and phosphorylation. Humans have >500 protein kinases and >12 targets have been established for anticancer drugs. These kinases are typically subdivided into tyrosine, serine/threonine, or both [37]. Via the inhibition by drugs of protein kinase activity in CRC cells, Qi et al. reported that protein kinase-inhibited cancer cells had decreased migration activity, destroyed cytoskeleton, increased apoptosis, and inhibited growth [38].

In the cytoHubba analysis, a redder color and more connections with other genes indicated that the core role of a gene was more prominent. Among the hub genes, *CCNB1*, *CCNA2*, *KIF14*, *TTK*, and *KIF20A* were most closely associated with other genes, and were possibly the most critical regulatory genes. *CCNB1* encodes regulatory proteins involved in mitosis and is required for the proper control of G2/M cell cycle transition. Inhibiting *CCNB1* expression in cancer cells inhibited cancer cell proliferation and induced cell cycle arrest and apoptosis in CRC and gastric cancer [39,40]. *CCNB1* was also closely related to ESC clinical prognosis [41]. *CCNA2* is involved in regulating the cell cycle by binding to cyclin-dependent kinases that promote G1/S and G2/M transitions. *CCNA2* was closely related to OC prognosis [42]. In mice, *CCNA2* deficiency increased DNA damage in colon mucosal epithelial cells, caused inflammation and increased cell proliferation and developmental abnormalities that increased colon cancer incidence [43]. In patients with liver cancer, the activation of *CCNA2* promoted liver cancer cell aggressiveness, where the patients also had worse prognosis [44]. The *KIF14* gene is a microtubulomokinin-driven protein-3 superfamily member involved in many biological processes. High *KIF14* expression was related to the GC stage and metastasis, where higher *KIF14* expression was related to stronger GC cell proliferation, invasion, and migration ability, and a lower survival rate in patients [45]. In ESC, downregulating *KIF14* inhibited esophageal squamous cell carcinoma cell proliferation, invasion, migration, and angiogenesis [46]. The *TTK* gene encodes a bi-specific protein kinase that can phosphorylate tyrosine, serine, and threonine. Failure to degrade this protein kinase leads to mitotic spindle abnormalities and tumorigenesis. The TTK protein is commonly overexpressed in PC, and the loss of the *TTK* gene inhibited cancer cell proliferation and transformation growth, where TTK was crucial in preventing PC cell death [47]. The protein encoded by *KIF20A* has protein kinase binding activity and regulates microbundle formation, midbody shedding, and cell division. *KIF20A* is related to the specific immune response of cholangiocarcinoma [48]. In liver cancer, deleting *KIF20A* expression inhibited HCC cell proliferation and enhanced HCC cell chemical sensitivity to cisplatin and sorafenib [49].

The transcription initiation process in eukaryotes often requires the assistance of various protein factors. Transcription factors and RNA polymerase Ⅱ form a transcription initiation complex and participate in the transcription initiation process together. In our study, the transcription factors E2F1, E2F3, E2F4, MYC, TP53, and YBX1 participated in the hub gene transcriptional regulation. E2F1, E2F3, and E2F4 are all transcription factor E2F family members, which are critical in controlling tumor-suppressor protein production and the cell cycle and are also targeted by small-DNA tumor virus transformation proteins. In vitro, different E2F1 levels exert other effects on cell fate: low E2F1 levels promote cell cycle progression, moderate E2F1 levels lead to cell cycle arrest, and high E2F1 levels lead to apoptosis [50]. The E2F family transcription factors are essential in GC pathogenesis [51]. In CRC, the combination of E2F1 and NCAPD3 promotes glycolysis, inhibits the tricarboxylic acid cycle, and participates in reprogramming glucose metabolism in CRC cells [52]. In HCC, the E2F4 expression level was positively correlated with tumor size, and E2F4 overexpression significantly enhances HCC cell proliferation, migration, and invasion [53]. In ESC, E2F3 overexpression induces RACGAP1 expression, thereby enhancing the cancer-promoting effect of RACGAP1 [54]. MYC is a transcription factor that activates or inhibits the transcription of target genes by regulating cell growth, apoptosis, differentiation, and metabolism through networks between proteins. Smoking and alcohol use cause genetic mutations in the proto-oncogene, *MYC*, which promotes oral cancer [55]. Furthermore, MYC can induce HMGA1 and TRIP13 transcription by bile duct cancer cells to enhance bile duct cancer cell migration, proliferation, invasion, dryness, and epithelial–mesenchymal transition (EMT) [56]. Tumor protein p53 (TP53) is a tumor-suppressor. The activation of wild-type *TP53* leads to cell cycle arrest, DNA repair, and cell death. TP53 inhibited *SLC7A11* transcription and promoted ferroptosis in PC cells [57]. In addition, *TP53* mutations are the most common mutation in HCC, affecting HCC progression and prognosis [58], and the same effect was observed in cholangiocarcinoma [59]. *YBX1* encodes proteins as both DNA- and RNA-binding proteins, participates in transcription and translation regulation and other processes, and is vital in microRNA processing. In CRC, targeting YBX1 phosphorylation might inhibit NF-κB activity in cancer, thereby regulating CRC cell growth [60]. Furthermore, GC development is inhibited after YBX1 is inhibited by the upstream circFAT1 (e2) [61].

## 5. Conclusions

Through multi-dimensional bioinformatics analysis and tumor tissue validation via in vitro experiments, we identified nine hub regulatory genes that participate in the pathogenesis of seven digestive system cancers and COVID-19. We explored the protein interactions between the genes and the regulatory role of transcription factors, providing novel insights for diagnosing and treating digestive system cancers and COVID-19. However, our study has some limitations, mainly regarding bioinformatics’ analysis. In vitro experiments were conducted to analyze gene expression differences. The interaction between the genes requires further study to clarify their mutual regulatory effects.

## Figures and Tables

**Table 1:** Basic information on seven cancers and COVID-19.

Disease	Oral Cancer	Esophageal Cancer	Gastric Cancer	Colorectal Cancer	Hepatocellular Cancer	Bile Duct Cancer	Pancreatic Cancer	COVID-19
GEO	GSE30784	GSE44021	GSE29272	GSE106582	GSE102079	GSE26566	GSE62165	GSE171110
Normal	45	73	134	117	105	65	13	10
Tumor/ COVID-19	167	73	134	77	152	104	118	44
Platform	GPL570	GPL571	GPL96	GPL10558	GPL570	GPL6104	GPL13667	GPL16791

**Table 2:** Common downregulated and upregulated genes.

Type	Up-Regulating						Downregulated
Gene	*CCNA2*	*CCNB1*	*CDC20*	*CDKN3*	*CEP55*	*COL1A2*	*NR3C2*
	*COL4A1*	*ECT2*	*IGF2BP3*	*KIF14*	*KIF20A*	*KIF4A*	
	*MELK*	*MMP9*	*NEK2*	*NUSAP1*	*PRC1*	*RAD51AP1*	
	*TPX2*	*TRIP13*	*TTK*	*UBE2C*			

**Table 3:** Basic information of the seven TCGA datasets.

Disease	Oral Cancer	Esophageal Cancer	Gastric Cancer	Colorectal Cancer	Hepatocellular Cancer	Bile Duct Cancer	Pancreatic Cancer
Normal	32	11	32	51	50	9	171
Tumor	330	163	375	647	374	35	179

**Table 4:** Basic information of seven cancer GEO datasets.

Disease	Oral Cancer	Esophageal + Gastric Cancer	Gastric Cancer	Colorectal Cancer	Hepatocellular Cancer	Bile Duct + Pancreatic Cancer	Pancreatic Cancer
GEO	GSE37991	GSE96668	GSE66229	GSE44076	GSE112790	GSE60979	GSE15471
Normal	40	11	100	148	15	12	39
Tumor	40	49	300	98	183	65	39
Platform	GPL6883	GPL10558	GPL570	GPL13667	GPL570	GPL14550	GPL570

**Table 5:** Basic data of blood GEO datasets.

Disease	Hepatocellular Cancer	Hepatocellular Cancer	Severe COVID-19	COVID-19
GEO	GSE114564 Normal/Tumor	GSE114564 Hepatitis/Tumor	GSE171110 Normal/severe COVID-19	GSE152418 Normal/COVID-19
Normal/ Hepatitis	15	20	10	17
Tumor/ COVID-19	63	63	44	17
Platform	GPL11154	GPL11154	GPL16791	GPL24676

**Table 6:** Regulatory relationship between transcription factors and hub genes.

Key TF	Description	*p* Value	List of Overlapped Genes
E2F1	E2F transcription factor 1	0.00174	*CCNB1*, *ECT2*
E2F3	E2F transcription factor 3	1.57 × 10^−5^	*CCNB1*, *CCNA2*
E2F4	E2F transcription factor 4, p107/p130-binding	5.09 × 10^−5^	*TTK*, *CCNB1*
MYC	v-myc myelocytomatosis viral oncogene homolog (avian)	0.000977	*CCNB1*, *CCNA2*
TP53	tumor protein p53	0.0026	*CCNA2*, *CCNB1*
YBX1	Y box binding protein 1	8.74 × 10^−5^	*CDKN3*, *CCNB1*

## Data Availability

The data that support the results of current study are available on TCGA, GEO and other databases. The datasets used and/or analyzed during the current study are available from the corresponding author on reasonable request.
